# An Ointment for Pruritis

**Published:** 1898-02

**Authors:** 


**An Ointment for Pruritis.**—The *Journal de Medicine de Paris* for October 3, attributes the following formula to Coover:

℞ Yellow oxide of mercury...................... 1 part
Vaseline........................................200 parts

M. The ointment should be applied at bedtime and also, if necessary, in the morning, by firm and prolonged friction, the affected parts having been previously washed with warm soap and water. It is said to allay the most intense itching.

